# First report of *Cryptosporidium andersoni* and risk factors associated with the occurrence of *Cryptosporidium* spp. in pre-weaned native Korean calves with diarrhea

**DOI:** 10.3389/fvets.2023.1145096

**Published:** 2023-03-21

**Authors:** Dong-Hun Jang, Hyung-Chul Cho, Yu-Jin Park, Jinho Park, Kyoung-Seong Choi

**Affiliations:** ^1^Department of Animal Science and Biotechnology, College of Ecology and Environmental Science, Kyungpook National University, Sangju, Republic of Korea; ^2^College of Veterinary Medicine, Jeonbuk National University, Iksan, Republic of Korea

**Keywords:** *Cryptosporidium* spp., diarrhea, pre-weaned calves, age, season

## Abstract

*Cryptosporidium* spp. are important enteric protozoan parasites that infect humans and other animals throughout the world. *Cryptosporidium* infection in cattle industry leads to substantial economic losses due to diarrhea, growth retardation, weight loss, and possibly death. Most studies have focused on *C. parvum*, and studies on other *Cryptosporidium* spp. and calf diarrhea are limited. Therefore, this study aimed to investigate the occurrence of *Cryptosporidium* spp. in pre-weaned calves, to determine the risk factors for *Cryptosporidium* spp. infection such as age and season, and to identify subtypes of *C. parvum* circulating in the Republic of Korea (ROK). A total of 510 fecal samples were collected from calves with diarrhea and divided by age and season. *Cryptosporidium* spp. were first screened using PCR targeting the small subunit (SSU) rRNA gene and further the 60-kDa glycoprotein gene for subtyping of *C. parvum*. Out of 510 fecal samples, 71 (13.9%) were positive for *Cryptosporidium* spp. in pre-weaned calves with diarrhea. *C. andersoni* (2.8%), *C. bovis* (30.9%)*, C. parvum* (29.6%), and *C. ryanae* (36.6%) were identified. *C. ryanae* was the most predominant in calves in the ROK. Calf age was a significant risk factor for *C. bovis* (χ^2^ = 13.83, *P* = 0.001)*, C. parvum* (χ^2^ = 7.57, *P* = 0.023), and *C. ryanae* (χ^2^ = 20.18, *P* = 0.000) occurrence. Additionally, *C. parvum* was detected 3.1-fold more frequently in pre-weaned calves with diarrhea in fall (95% CI: 1.23–7.81; *P* = 0.016) than in spring, whereas *C. ryanae* was 8.9-fold more frequently detected in summer (95% CI: 1.65–48.68; *P* = 0.011) than in spring. Three subtypes (IIaA17G4R1, IIaA18G3R1, and IIaA20G3R1) of *C. parvum* were identified. Of them, IIaA17G4R1 was the most common, whereas IIaA20G3R1 was not previously detected in calves in the ROK. To our knowledge, this is the first report of *C. andersoni* in pre-weaned calves in the ROK. The occurrence of *Cryptosporidium* spp. appears to be age-dependent in calves. Season had a significant effect on the occurrence of *C. parvum* and *C. ryanae*. Taken together, *C. bovis* and *C. ryanae* along with *C. parvum* are detected in pre-weaned calves with diarrhea and these two pathogens should not be overlooked in the diagnosis of calf diarrhea.

## 1. Introduction

*Cryptosporidium* spp. are important protozoan parasites that affect the gastrointestinal tract in various animals, including humans. *Cryptosporidium*, with *Giardia*, is known as representative water-borne pathogen in humans (1–3). Infection with these species occurs *via* the fecal-oral route, either by direct contact with infected animals or by ingestion of infective oocysts from contaminated water and/or food (4). *Cryptosporidium* is one of the major pathogens causing diarrhea in neonatal calves, which is characterized by watery and sometimes bloody stool, loss of appetite, abdominal pain, growth retardation, weight loss, and possibly death, resulting in substantial economic losses (5–11).

To date, 44 *Cryptosporidium* spp. have been identified (12), and among them, four species, namely, *C. andersoni, C. bovis, C. parvum*, and *C. ryanae*, have been identified in cattle. Their prevalence has been shown to have an age-related distribution: *C. parvum* in pre-weaned (13), *C. ryanae* and *C. bovis* in post-weaned (14), and *C. andersoni* in yearlings and adult cattle (15). In addition, *C. andersoni* has been found in all age groups (16, 17). However, the occurrence of these species is not necessarily age-related, and a few studies performed in several countries have reported different patterns of infection (13, 16, 18–20). *C. bovis, C. parvum*, and *C. ryanae* are bovine intestinal species and often cause atrophy of the villi, shortening of the microvilli, and destruction of the intestine, resulting in diarrhea, whereas *C. andersoni* infects the abomasum, which causes gastritis (5, 17, 21). Most importantly, *C. andersoni, C. bovis*, and *C. ryanae* are associated with subclinical infection in cattle (22–24). Molecular methods should be used for species differentiation because of their indistinguishable sizes and shapes (24, 25).

*C. parvum* accounts for over 90% of *Cryptosporidium* infections in neonatal calves (26). Calves with diarrhea were reportedly 36.5 times more likely to be infected with *C. parvum* than calves without diarrhea (17). *C. parvum* is the most prevalent species infecting humans and calves worldwide, whereas *C. andersoni* and *C. bovis* are rarely found in humans (4, 23, 27). Recently, *C. bovis* and *C. ryanae* have been identified in pre-weaned calves (28–31); however, their pathogenicity and role in calf diarrhea remain unclear. According to a previous study performed by our group, *C. ryanae* was detected at a higher frequency in calves with diarrhea than *C. bovis* and has now emerged as a leading cause of diarrhea alongside *C. parvum* (31). *C. parvum* subtypes have been detected using the 60-kDa glycoprotein (*gp60*) gene, and to date, at least 14 families (IIa and IIo) have been identified (12, 27). Among them, two families, IIa and IId, are found in both humans and ruminants, and are known as having zoonotic potential. Family IIa has been identified in calves in most industrialized nations (4), and IIaA15G2R1 is the most dominant subtype reported in cattle worldwide (27). Additionally, family IId has been found in dairy calves in some countries (20, 32–36). In contrast, pre-weaned calves with diarrhea in the Republic of Korea (ROK) are mostly infected with *C. parvum*, almost exclusively with family IIa, especially IIaA18G3R1 (31). *C. parvum* oocysts in water flowing out of stables contaminate the surrounding agricultural water or infect humans or wildlife. Hence, infected cattle pose a potential threat to human health owing to environmental contamination by excreted oocysts (37).

Currently, most studies have focused on *C. parvum*, and reports on the association between other *Cryptosporidium* spp. and calf diarrhea are limited. Therefore, the objective of this study was to investigate the positivity of *Cryptosporidium* spp. in pre-weaned calves with diarrhea in the Republic of Korea (ROK), to identify *Cryptosporidium* spp. that cause diarrhea according to age group, to assess the association between age or season and *Cryptosporidium* spp., and to determine *C. parvum* subtypes circulating in calves, which act as major risk factors for zoonotic infection.

## 2. Materials and methods

### 2.1. Ethics statement

All animal procedures were conducted according to ethical guidelines for the use of animal samples, and were approved by the Jeonbuk National University (Institutional Animal Care and Use Committee Decision No. JBNU 2020-052).

### 2.2. Sample collection and DNA extraction

Between January 2021 and May 2022, a diarrhea outbreak was noticed on 181 farms in four different provinces (Gyeonggi, Gyeongbuk, Gyeongnam, and Jeonbuk) in the ROK. A total of 510 fresh fecal samples were collected directly from the rectum of individual diarrheic pre-weaned calves by a veterinarian using sterile plastic gloves. The majority of calves (*n* = 497) were of the Korean native breed (Hanwoo), while 13 were of the Holstein breed. All these calves were maintained indoors. The collected samples were placed in sterile plastic tubes and immediately transported to the laboratory for subsequent DNA extraction. During sampling, age, sex, sampling date, fecal consistency, and farm location were recorded. All samples were stored at 4°C until DNA extraction. Fecal samples were divided according to calf age as follows: 1–10 days (*n* = 251), 11–30 days (*n* = 205), and 31–70 days (*n* = 54). To investigate the association between the occurrence of *Cryptosporidium* spp. and season, stool samples were classified into four groups: spring (March–May; *n* = 206), summer (June–August; *n* = 80), fall (September–November; *n* = 158), and winter (December–February; *n* = 66). The average temperature and rainfall for each season were as follows: spring (13.0 ± 4.3°C, 72.4 ± 39.6 mm), summer (24.6 ± 2.0°C, 247.7 ± 117.8 mm), fall (15.6 ± 6.6°C, 97.2 ± 58.5 mm), and winter (0.7 ± 1.8°C, 14.0 ± 9.7 mm).

### 2.3. PCR amplification and sequencing

Genomic DNA was extracted from 200 mg of each fecal sample using an AccuPrep Stool DNA extraction kit (Bioneer, Daejeon, ROK) according to the manufacturer's instructions. Extracted DNA was stored at −20°C until use. *Cryptosporidium* spp. was first identified using the small subunit (SSU) rRNA gene by nested polymerase chain reaction (PCR) (38). Next, *C. parvum* was screened by targeting the 60-kDa glycoprotein (*gp60*) gene using nested PCR to determine its subtype (39, 40). Primers used in this study are listed in Table 1. The PCR conditions were as follows: 94°C for 3 min, followed by 35 cycles at 94°C for 45 s, annealing at 54°C for 45 s, 72°C for 60 s, and a final extension at 72°C for 7 min. Negative controls were included in all runs. Amplified PCR products were sepa-rated by electrophoresis on a 1.5% agarose gel and visual-ized after staining with ethidium bromide. All secondary PCR products were purified using an AccuPower PCR Purification Kit (Bioneer, Daejeon, ROK) and directly sequenced (Macrogen, Daejeon, ROK). PCR amplicons were sequenced using a BigDye Terminator 3.1 Cycle Sequencing Kit on a 3500 xL Genetic Analyzer (Applied Biosystems, Foster City, CA, USA), according to the manufacturer's instructions, using the same primer set as in the conventional PCR reaction. Among the PCR-positive samples, only samples with good sequencing results were considered positive for *Cryptosporidium* spp. The nucleotide sequences obtained in this study were analyzed using BioEdit (version 7.2.5) and compared with reference sequences using the Basic Local Alignment Search Tool available at the National Center for Biotechnology Information database (http://www.ncbi.nlm.nih.gov). The sequences of *C. bovis, C. ryanae*, and *C. andersoni* were compared using Geneious Prime software (version 2021.1.1; https://www.geneious.com) and analyzed *via* direct comparison with reference sequences from GenBank. The subtypes of *gp60* were named based on the repeated numbers of TCA-(A), TCG- (G), and ACATCA-(R), as described previously (41). All nucleotide sequences generated in this study were deposited in the GenBank database under the following accession numbers; OQ001482–OQ001483 for *C. andersoni*, OQ001456–OQ001477 for *C. bovis*, OQ001429–OQ001454 for *C. ryanae*, and OQ025024–OQ025044 for *C. parvum*.

**Table 1:** Primers used for the detection of *Cryptosporidium* spp.

**Target genes**	**Primer names**	**Sequences (5^′^-3^′^)**	**Annealing temp (°C)**	**Amplicon size (bp)**
SSU rRNA	F	TTCTAGAGCTAATACATGCG	54	1325
	R	CCCATTTCCTTCGAAACAGGA		
	F2	GGAAGGGTTGTATTTATTAGATAAAG	54	830
	R2	AAGGAGTAAGGAACAACCTCCA		
*gp60*	F	ATAGTCTCCGCTGTATTC	52	980–1000
	R	GCAGAGGAACCAGCATC		
	F2	TCCGCTGTATTCTCAGCC	52	467
	R2	GAGATATATCTTGGTGCG		

### 2.4. Statistical analysis

The statistical analysis was performed using the SPSS Statistics 26 software package for Windows (SPSS Inc, Chicago, IL, USA). A chi-square test was determined the association between the occurrence of each *Cryptosporidium* spp. and age or season. We constructed a generalized linear mixed model (GLMM) for a binomial family, with a logit link function for each *Cryptosporidium* species to test whether age and season affected the infection. In the model, the origin of farm and sampling year were considered as random effects. Odd ratios (OR) with 95% confidence intervals (CIs) were calculated to assess the association between age or season and occurrence of *Cryptosporidium* spp. The correlation between precipitation and occurrence was confirmed through correlation analysis. A *P-*value < 0.05 was considered statistically significant.

## 3. Results

### 3.1. Detection of *Cryptosporidium* spp.

Of the 510 fecal samples, 71 (13.9%) were positive for *Cryptosporidium* spp. as determined by PCR analysis using the SSU rRNA gene. Four species were identified in the pre-weaned Korean native calves with diarrhea (Table 2; Supplementary Figure 1), of which *C. ryanae* was the most predominant species (36.6%, 26/71), followed by *C. bovis* (30.9%, 22/71), *C. parvum* (29.6%, 21/71), and *C. andersoni* (2.8%, 2/71). Co-infections with two or three species were not observed.

**Table 2:** Positivity of *Cryptosporidium* spp. according to age group in pre-weaned calves with diarrhea in the ROK.

**Age groups (days)**	**No. of positive samples**	***Cryptosporidium*** **species (*****n*****)**			
		***C. andersoni***	***C. bovis***	***C. parvum***	***C. ryanae***
1–10 (*n* = 251)	17 (6.8%)	0	3	9	5
11–30 (*n* = 205)	37 (18.0%)	2	17	6	12
31–70 (*n* = 54)	17 (31.5%)	0	2	6	9
Total (*n* = 510)	71 (13.9%)	2	22	21	26

ROK, Republic of Korea.

### 3.2. Distribution of *Cryptosporidium* spp. by age

The occurrence of *Cryptosporidium* spp. in calves showed distinct characteristics according to age (Table 2). *C. bovis, C. parvum*, and *C. ryanae* were detected in all the age groups. Positivity of *C. bovis* was the highest in calves aged 11–30 days, whereas positivity of *C. ryanae* gradually increased with age and peaked in calves aged 31–70 days. Positivity of *C. parvum* were the highest in calves aged 31–70 days. *C. andersoni* was found in only two calves aged 11–30 days. Except for *C. andersoni, C. bovis* (χ^2^ = 13.825, *P* = 0.001), *C. parvum* (χ^2^ = 7.570, *P* = 0.023), and *C. ryanae* (χ^2^ = 20.184, *P* = 0.000) was significantly associated with the age of the calves. Thus, the risk of contracting *C. bovis* and *C. parvum* was 7.8-fold higher in calves aged 11–30 days (95% confidence interval [CI]: 2.25–27.04; *P* = 0.001) and 4.2-fold higher in calves aged 31–70 days (95% CI: 1.42–12.54; *P* = 0.010), respectively, than in calves aged 1–10 days (Table 3). Moreover, the risk of contracting *C. ryanae* was 3.30-fold higher in calves aged 11–30 days and 11.38-fold higher in calves aged 31–70 days (95% CI: 3.62–35.84; *P* = 0.000) than in calves aged 1–10 days (Table 3).

**Table 3:** Generalized linear mixed model results for *Cryptosporidium* spp. occurrence in pre-weaned calves with diarrhea in the ROK.

**Species**	**Fixed effects**		**OR**	**95% CI**	***P*-value**
	**Factors**	**Level**			
*C. bovis*	Age	1–10 (Ref.)	–	–	–
		11–30	7.800	2.250–27.039	0.001^*^
		31–70	4.216	0.681–26.087	0.122
	Season	Spring (Ref.)	–	–	–
		Summer	–	–	–
		Fall	1.299	0.293–5.748	0.730
		Winter	1.122	0.328–3.836	0.854
*C. parvum*	Age	1–10 (Ref.)	–	–	–
		11–30	0.918	0.321–2.627	0.873
		31–70	4.216	1.141–12.536	0.010^*^
	Season	Spring (Ref.)	–	–	–
		Summer	0.766	0.171–3.432	0.727
		Fall	3.106	1.234–7.818	0.016^*^
		Winter	1.018	0.233–4.440	0.981
*C. ryanae*	Age	1–10 (Ref.)	–	–	–
		11–30	3.304	1.142–9.552	0.027^*^
		31–70	11.384	3.616–5.841	0.000^*^
	Season	Spring (Ref.)	–	–	–
		Summer	8.974	1.654–48.683	0.011^*^
		Fall	4.240	0.811–22.155	0.087
		Winter	1.378	0.255–7.444	0.709

OR, odds ratio; CI, confidence interval; ^*^significant.

### 3.3. Seasonal distribution of *Cryptosporidium* spp.

**Figure 1:**
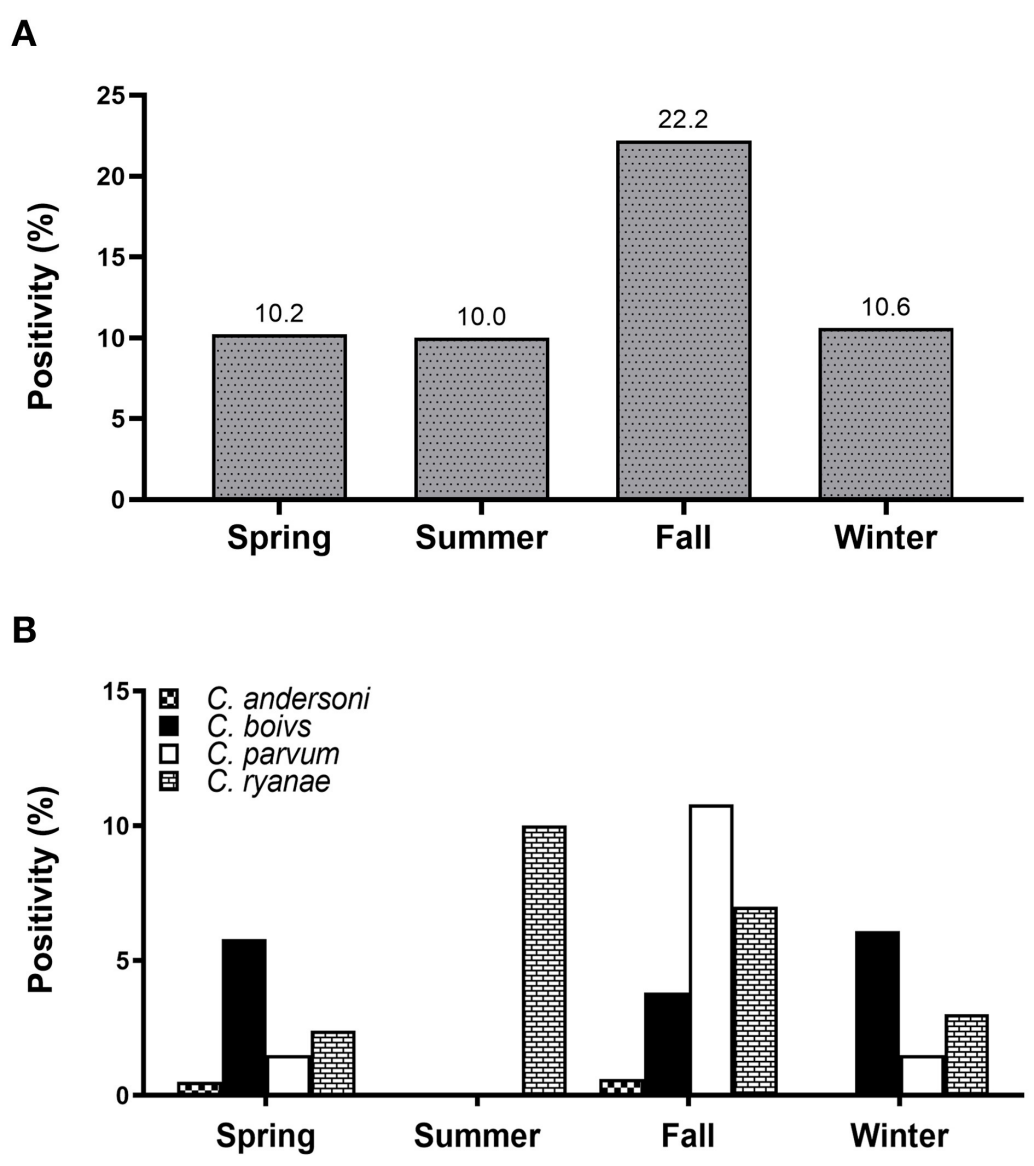
Seasonal positivity of *Cryptosporidium* spp. in pre-weaned native Korean calves with diarrhea **(A)**. Distribution of four *Cryptosporidium* spp. in calves according to the season **(B)**.

All four *Cryptosporidium* spp. were detected in spring and fall. Positivity of *Cryptosporidium* spp. was the highest in fall (22.2%), and there were no differences in the occurrence in any other season (Figure 1A). Of the four species, *C. ryanae* was found in all seasons and, unlike the other species, was exclusively detected in summer (Figure 1B). The GLMM approach was used to evaluate the association between the occurrence of *Cryptosporidium* spp. and season. The results showed that the occurrence of *C. parvum* and *C. ryanae* was significantly associated with season. *C. parvum* was detected 3.1-fold more frequently in pre-weaned calves with diarrhea in fall (95% CI: 1.23–7.81; *P* = 0.016) than in spring. *C. ryanae* was 8.9-fold more frequently detected in summer (95% CI: 1.65–48.68; *P* = 0.011) than in spring (Table 3). Although positivity of *C. bovis* was relatively high in calves in spring and winter, there was no statistically significant difference between seasons (*P* = 0.149).

### 3.4. *C. parvum* subtyping

Among 21 *C. parvum*-positive samples, three subtypes were identified using the *gp60* gene (Supplementary Table 1). IIaA17G4R1 (66.7%, 14/21) was the predominant subtype, followed by IIaA18G3R1 (19.0%, 4/21), and IIaA20G3R1 (14.3%, 3/21). IIaA17G4R1 and IIaA18G3R1 were detected in pre-weaned calves of all age groups, whereas the IIaA20G3R1 subtype was identified only in calves younger than 30 days.

## 4. Discussion

In the present study, we identified different *Cryptosporidium* spp. in pre-weaned calves with diarrhea, with a 13.9% positivity, which is higher than that reported previously in the ROK (42–44). The main reason for the difference is that past research has focused mainly on *C. parvum*, and the occurrence of other *Cryptosporidium* spp. has recently increased (29–31, 45, 46). Compared with a previous study (31), the current results showed that the occurrence of *C. bovis* and *C. ryanae* have increased noticeably in calves. *C. bovis, C. parvum*, and *C. ryanae*, but not *C. andersoni*, were detected at similar rates in pre-weaned calves. Although *C. bovis* and *C. ryanae* were detected in calves with diarrhea in this study, the role and clinical significance of these species in calf diarrhea remain unclear. Thus, these findings suggest that in addition to *C. parvum, C. bovis* and *C. ryanae* might be representative species that infect young calves. Notably, this is the first report of *C. andersoni* infection and the first time that the IIaA20G3R1 subtype of *C. parvum* has been described in pre-weaned calves with diarrhea in the ROK.

Four *Cryptosporidium* species were identified in pre-weaned calves with diarrhea: *C. andersoni, C. bovis, C. parvum*, and *C. ryanae*. In the ROK, *C. andersoni* infection was detected in calves aged 11–30 days for the first time. *C. andersoni* is more frequently found in post-weaned or adult cattle (17, 47), and its infection causes decreased milk production and poor weight gain (48–50). In particular, *C. andersoni* has been observed in cattle of all age groups and was reported to be the second most prevalent species in pre-weaned calves in China (51). However, to date, this has not been reported in cattle in the ROK. Although in this study, *C. andersoni* infection was found in only two calves, our results demonstrate that young calves can acquire *C. andersoni* infection at an early stage of life (20, 52). *C. andersoni* causes diarrhea in humans and is one of the main *Cryptosporidium* species found in contaminated water (53, 54). Moreover, no information is available on the occurrence of *C. andersoni* and its association with diarrhea in humans in the ROK. Thus, we cannot draw a conclusion regarding the infection route of *C. andersoni* in these calves. However, the possibility that the calves may have been infected through the ingestion of water contaminated with oocysts shed by humans infected with *C. andersoni* cannot be completely ruled out. These results suggest that young calves are more susceptible to *Cryptosporidium* spp. and may act as an infection source for other animals. Further studies are thus required to investigate the occurrence and symptoms of *C. andersoni* in pre-weaned calves in the ROK.

According to our results, *C. ryanae* was mostly detected in pre-weaned native Korean calves. These results differ from those of a previous study (31). The reason for the difference may be related to the calf management system and method used for diagnosis. Our findings revealed that positivity of *C. ryanae* increased with age and was highest in calves older than 31 days. Our results indicated much higher occurrence with *C. ryanae* (36.6%) than reported previously (8.6%) for calves of this age in the ROK (31). *C. ryanae* infection in the ROK showed a continuous increase in pre-weaned calves. Compared with early neonatal calves, the risk of contracting *C. ryanae* in pre-weaned calves increased significantly with age. The reason for the high occurrence of *C. ryanae* in this study is not clear. It is speculated that *C. parvum* may affect the occurrence of *C. ryanae* and *C. bovis* in calves, and these two species may occur early on farms with little or no *C. parvum* infection, resulting in increased occurrence. Another possibility is that the oocysts of *C. ryanae* may be more resistant to adverse environmental conditions than those of other species and survive well in high temperatures and moist conditions (55). Therefore, the infection may last for weeks or months. In addition, *C. ryanae* is associated with the occurrence of severe diarrhea in calves (28, 56). However, several studies have shown that *C. ryanae* is less frequently associated with diarrhea (24, 30, 45). However, the findings of this study indicated that *C. ryanae* was detected in pre-weaned calves with diarrhea, and calves older than 31 days were at higher risk.

*C. bovis* was the second most dominant species in pre-weaned calves with diarrhea in the ROK. This is in contrast to other studies in which *C. bovis* was the most common species (20, 30, 51, 57, 58) which demonstrates the differences in the occurrence of *Cryptosporidium* species between countries. In relation to age, positivity of *C. bovis* was highest in calves aged 11–30 day. This result is consistent with those of previous studies (59–61). *C. bovis* has also been reported in pre-weaned calves and post-weaned calves (18, 30, 45, 62) and is associated with the occurrence of severe diarrhea in calves (63, 64). However, several studies have reported that *C. bovis* does not cause diarrhea, similar to studies of *C. ryanae* (18, 29, 30, 45, 65). To date, studies on the occurrence and clinical symptoms of *C. bovis* in calves in the ROK are considerably scarce (31, 43) owing to the rarity of this infection in calves. Although *C. bovis* was detected in the diarrheic calves, currently, it cannot be asserted whether the observed diarrhea was caused by *C. bovis*. Accordingly, the results suggest that calves aged 11–30 days might be at high risk for *C. bovis* infection. Therefore, further research should be conducted to elucidate the pathogenicity of *C. bovis* and *C. ryanae* in calf diarrhea.

Interestingly, positivity of *C. parvum* in this study was the third lowest in pre-weaned calves in the ROK. Contrary to previous studies which showed that *C. parvum* mainly infects young calves (15, 59, 60, 66), the present results showed that the positivity of *C. parvum* were highest in calves aged 31–70 days, whereas those in calves under 30 days of age were very low. In general, *C. parvum* infection starts soon after birth, peaks at 2–3 weeks of age, and recovers mostly by 4 weeks (45, 65–68). The results of the present study are inconsistent with those of our previous studies, which showed no occurrence in calves aged 31–70 days (31, 42). The reason for this discrepancy cannot be explained at this time, but might be due to several factors, such as crowding, inadequate disinfection of the barn floor, environmental contamination, or the presence of other hosts or wildlife. Unfortunately, we did not consider any of these factors. Several studies have shown that *C. parvum* infection occurs mostly in calves aged between 31 days and 6 months (15, 17, 28). These results are somewhat consistent with ours. Wells et al. (69) reported that 80% of adult cattle were shedding *C. parvum* (69). This implies that adult cattle are likely a source of *C. parvum* infection in calves. Although *C. parvum* has been regarded as a major pathogen in young calves, it has not been extensively examined in post-weaning calves and adult cattle in the ROK. Considering the high positivity of *C. parvum* in calves aged 31–70 days, the possibility of re-infection from their dams cannot be excluded. Thus, *C. parvum* infection in calves appears to be more affected by the environment in which the calves are raised than by their age.

In the present study, we found a seasonal association in the occurrence of *Cryptosporidium* spp. Our results revealed that season had a significant effect on the occurrence of *C. parvum* and *C. ryanae*: *C. parvum* dominance in fall, and *C. ryanae* dominance in summer. However, this finding differs from previous results, which reported that the occurrence of *C. bovis* was dominant in summer, whereas that of *C. parvum* was dominant in spring and winter (58, 70). According to another study performed in the ROK, season and the occurrence of *C. parvum* were not associated (43). The relationship between season and the occurrence of *Cryptosporidium* spp. remains unclear, but it seems that their occurrence may vary depending on the farm situation in each country. Notably, levels of sunlight, temperature, humidity, and precipitation, as well as breeding density, may play a role in this variation (71, 72). One possibility is that calves are mostly born from spring to summer in the ROK, which increases the density of calves within the herd and makes calves more susceptible to *Cryptosporidium* spp. oocysts shed by older calves that were born earlier in the season. Especially, in the ROK, it rains heavily in summer. Heavy rain results in the overcrowding of animals and hampers the drying of the floor, thereby increasing the survival of *Cryptosporidium* oocysts (71). Our results showed that precipitation in the preceding month was significantly associated with the occurrence of *Cryptosporidium* spp. (*P* = 0.015) (data not shown). As previously mentioned, precipitation is highest in summer, followed by that in fall, spring, and winter. *Cryptosporidium* spp. oocysts are likely to easily spread through rivers or groundwater to surrounding farms due to heavy rain, which increases the opportunity for calves to be exposed. Owing to the long survival of *Cryptosporidium* spp. oocysts, susceptible calves with lower immunity are more frequently present from summer to fall. These results suggest that precipitation may act as an important factor in the occurrence of cryptosporidiosis. Our findings provide new insights on the seasonal occurrence of *Cryptosporidium* spp., and hence, their control.

Subtype analysis of *C. parvum-*positive samples revealed the presence of three subtypes: IIaA17G4R1, IIaA18G3R1, and IIaA20G3R1. Of these, IIaA17G4R1 was the most dominant subtype in pre-weaned calves with diarrhea in the ROK. Our results differed from those of a previous study in which IIaA18G3R1 was the predominant subtype in pre-weaned calves in the ROK (31). Contrary to a previous report (31) that found 11 subtypes, only three subtypes were identified in this study. This was mainly because of the difference in the number of positive samples. However, this could be explained by the decreased movement of humans or animals due to the COVID-19 pandemic. The IIaA17G4R1 subtype has been identified in calves and goats in several countries (31, 73–76); however, its age-related distribution in calves remains unclear. Nevertheless, IIaA17G4R1 was detected in all age groups in the present study. As mentioned earlier, the IIaA17G4R1 subtype may be shed from adult cattle and re-infected calves. Moreover, the IIaA20G3R1 subtype has been detected in humans and cattle (3, 77), and was identified for the first time in calves younger than 1 month in the ROK. This subtype is considered to be more zoonotic than IIaA17G4R1. Thus, calves may be major contributors to human cryptosporidiosis.

## 5. Conclusions

The study confirmed the presence of four *Cryptosporidium* spp., *C. andersoni, C. bovis, C. parvum*, and *C. ryanae* in pre-weaned calves with diarrhea, with *C. ryanae* being the most predominant species and IIaA17G4R1 being the most predominant subtype of *C. parvum* in the ROK. To the best of our knowledge, this is the first study to report a *C. andersoni* infection and a new subtype, IIaA20G3R1 of *C. parvum*, in calves. The occurrence of *Cryptosporidium* spp. was significantly associated with the season, *C. parvum* being dominant in fall and *C. ryanae* dominant in summer. Although the current study did not elucidate the association between *C. bovis* and *C. ryanae* and diarrhea, these findings improved our understanding of the epidemiology and control of bovine cryptosporidiosis in the ROK. Further research is required to investigate the association between *C. bovis* and *C. ryanae* and diarrhea according to fecal consistency and identify the risk factors affecting *Cryptosporidium* infection in calves.

## Data availability statement

The datasets presented in this study can be found in online repositories. The names of the repository/repositories and accession number(s) can be found in the article/Supplementary material.

## Ethics statement

The animal study was reviewed and approved by the Jeonbuk National University (Institutional Animal Care and Use Committee Decision No. JBNU 2020-052). Written informed consent was obtained from the owners for the participation of their animals in this study.

## Author contributions

KSC and JP conceived the study. DHJ, HCC, YJP, and JP performed the experiments. DHJ, HCC, and KSC analyzed the data and wrote the manuscript. All authors read and approved the final manuscript.

## Supplementary material

Supplementary Figure 1 Nested PCR targeting SSU rRNA and *gp60* genes. Amplified PCR products were sepa-rated by electrophoresis on a 1.5% agarose gel and visual-ized after staining with ethidium bromide. Lane 1: DNA ladder (1 kb); Lanes 2–5: PCR amplicons for SSU rRNA (830 bp); Lanes 6 and 7: PCR amplicons for *gp60* (467 bp); and Lane 8: negative control.

Supplementary Table 1 Distribution of *C. parvum* subtypes in pre-weaned calves with diarrhea in the ROK.
