# Supplementary figures and images for: First report of Cryptosporidium andersoni and risk factors associated with the occurrence of Cryptosporidium spp. in pre-weaned native Korean calves with diarrhea
Source: Front Vet Sci. 2023 Mar 21;10:1145096. doi: 10.3389/fvets.2023.1145096 (PMC10070877; doi:10.3389/fvets.2023.1145096)

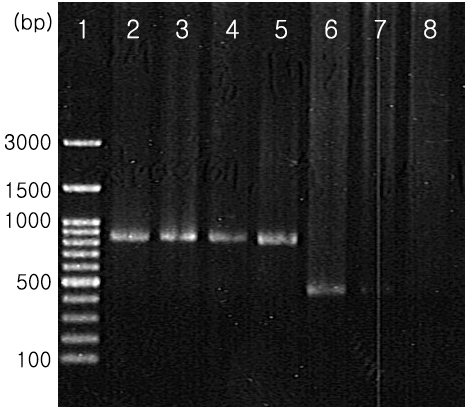

Supplement: Supplementary Figure 1 — Nested PCR targeting SSU rRNA and gp60 genes. Amplified PCR products were sepa-rated by electrophoresis on a 1.5% agarose gel and visual-ized after staining with ethidium bromide. Lane 1: DNA ladder (1 kb); Lanes 2–5: PCR amplicons for SSU rRNA (830 bp); Lanes 6 and 7: PCR amplicons for gp60 (467 bp); and Lane 8: negative control. [file Image_1.JPEG]
