# Supplementary material for: First report of Cryptosporidium andersoni and risk factors associated with the occurrence of Cryptosporidium spp. in pre-weaned native Korean calves with diarrhea
Source: Front Vet Sci. 2023 Mar 21;10:1145096. doi: 10.3389/fvets.2023.1145096 (PMC10070877; doi:10.3389/fvets.2023.1145096)
Supplement: Supplementary Table 1 — Distribution of C. parvum subtypes in pre-weaned calves with diarrhea in the ROK. [file Table_1.docx]

Supplementary Table 1. Distribution of *C. parvum* subtypes in pre-weaned calves with diarrhea in the ROK

| gp60 subtype | 1−10 days | 11−30 days | 31−70 days | Total |
| --- | --- | --- | --- | --- |
| IIaA17G4R1 | 5 | 4 | 5 | 14 |
| IIaA18G3R1 | 2 | 1 | 1 | 4 |
| IIaA20G3R1 | 2 | 1 | 0 | 3 |
